# Imbalanced Nutrient Intake in Cancer Survivors from the Examination from the Nationwide Health Examination Center-Based Cohort

**DOI:** 10.3390/nu10020212

**Published:** 2018-02-14

**Authors:** Boyoung Park, Jinhee Lee, Jeongseon Kim

**Affiliations:** National Cancer Center Graduate School of Cancer Science and Policy, 323 Ilsan-ro, Ilsandong-gu, Goyang-si, Gyeonggi-do 10408, Korea; hayejine@ncc.re.kr (B.P.); marie0715@naver.com (J.L.)

**Keywords:** cancer survivors, nutrient intake, high proportion of energy from carbohydrates, undernutrition

## Abstract

This study was conducted to examine the nutrient intake status of cancer survivors. A total of 5224 cancer survivors, 19,926 non-cancer individuals without comorbidities (non-cancer I), and 20,622 non-cancer individuals with comorbidities, matched by age, gender, and recruitment center location were included in the analysis. Generally, the proportion of total energy from carbohydrates was higher and the proportion from fat was lower in cancer survivors. The odds ratios (ORs) for total energy (OR = 0.92, 95% confidence interval (CI) = 0.86–0.99), proportion of total energy from fat (OR = 0.54, 95% CI = 0.35–0.83), and protein (OR = 0.85, 95% CI = 0.79–0.90) were significantly lower, and the OR for the proportion of total energy from carbohydrates was higher (OR = 1.21, 95% CI = 1.10–1.33) in the cancer survivors than in non-cancer I. Additionally, the cancer survivors’ protein, vitamin B_1_, vitamin B_2_, niacin, and phosphorus intakes were lower, whereas their vitamin C intake was higher. When divided by cancer type, the ORs for the carbohydrate percentages were significantly higher in the colon and breast cancer survivors, whereas protein intake was lower in gastric, breast, and cervical cancer survivors. The nutrient intake patterns in Asian cancer survivors are poor, with higher carbohydrate and lower fat and protein intakes.

## 1. Introduction

The number of cancer survivors has rapidly increased with the increases in both cancer incidence and survival rates over the last several decades [1], with 32.5 million 5-year cancer survivors worldwide [2]. In Korea, the 5-year cancer relative survival rate has improved compared with the rate 15 years ago, from 41% to 68%. The prevalence of cancer survivors was 2453 per 100,000 individuals in 2012. Compared with Western countries, the proportion of cancer survivors of gastric and thyroid cancer was higher due to higher incidence and relatively lower 5-year mortality rate [3]. Cancer survivors have an increased risk of not only secondary cancer but also other chronic diseases, including cardiovascular disease, endocrine diseases, and diabetes [4,5]. These adverse outcomes following cancer may be caused by the cancer itself, the treatments [6] or common modifiable lifestyle behaviors, suggesting the importance of informed lifestyle choices for cancer survivors [7,8,9]. Among the modifiable factors, nutrition or dietary habits play an important role not only in the development of cancer [10,11], but also in the etiology of chronic diseases, quality of life, or prognosis, including mortality from cardiovascular diseases in cancer survivors after cancer diagnosis [9,12]. Thus, identifying nutritional status and patterns might be a priority for cancer survivors, to improve long-term health and survival by planning and conducting appropriate nutritional interventions in these populations.

However, the few studies in Western countries that have focused on the adherence of cancer survivors to dietary guidelines have shown poor adherence in cancer survivors [8,13]. Given the diverse dietary patterns [14] and different cancer patterns between countries, regions, and ethnicities [15], the nutritional status in cancer survivors needs to be investigated in Asian countries. However, these studies are lacking.

Therefore, we conducted this study to evaluate the extent of adherence of cancer survivors to the dietary reference intakes for Koreans, compared to people without experience with cancer. Considering the emerging concept that cancer should be considered a chronic disease [16,17], we divided the people without experience with cancer into groups based on the presence or absence of comorbidities. Additionally, we evaluated the nutrient intake status by cancer type to identify whether dietary intake in cancer survivors was affected by cancer type.

## 2. Materials and Methods

### 2.1. Data Source and Study Population

This study used baseline survey data from a nationwide health examination center-based cohort (the Health Examinee (HEXA) cohort). The HEXA cohort is one part of the Korea Genome Epidemiology Study (KoGES), which is an ongoing population-based cohort study that began in 2001. In the HEXA cohort, the participants were recruited from health examination centers for regular check-ups in 14 Korean urban areas. The health examinees were aged 40–79 years, gave informed consent, and were asked to complete an interviewer-administered questionnaire that included sociodemographic factors, past medical history and family history, behavioral characteristics, and a validated food frequency questionnaire designed for Koreans. Additionally, the results of clinical tests and physical examinations from medical check-ups were collected. Details of the KoGES and HEXA cohort are described elsewhere [18,19] and on the Korea National Institute of Health website [20].

**Figure 1:**
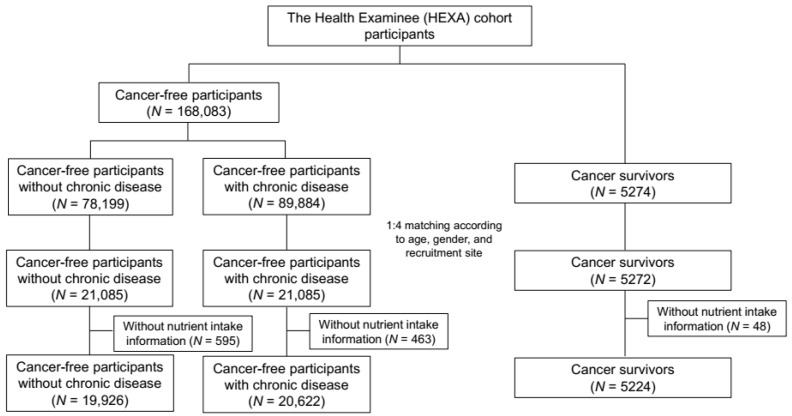
Flow chart of the study participants selection process.

Based on information from the past medical history collected in the baseline questionnaire, the cancer survivors were individuals who responded that they had been diagnosed with any type of cancer by a doctor, using the definition of DeSantis et al., who defined cancer survivors as any person who had been diagnosed with any type of cancer, including both patients under treatment and patients who had completely recovered [6]. A total of 5274 cancer survivors were identified. Participants without a self-reported cancer history were divided into two groups, as follows: non-cancer individuals without comorbidities (non-cancer I) and non-cancer individuals with comorbidities (non-cancer II). The collected comorbidities through the questionnaire included hypertension, diabetes, dyslipidemia, stroke, angina or myocardial infarction, gastrointestinal disease, intestinal polyps, fatty liver, chronic liver disease or liver cirrhosis, gallbladder, respiratory disease, thyroid disease, arthritis, osteoporosis, gout, glaucoma or cataracts and depression, and those who reported that they had been diagnosed with any type of above diseases by a doctor were considered non-cancer II group. This selection resulted in 78,199 subjects in the non-cancer I group and 89,884 subjects in the non-cancer II group. Through individual matching by age (±2 years), gender (male and female), and recruitment health examination location (39 centers) with a 1:4 ratio, 5272 cancer survivors, 21,085 people in non-cancer I, and 21,085 people in non-cancer II were included in the analysis. (Figure 1). The Institutional Review Board of the National Cancer Center approved this study protocol, which was in compliance with the Declaration of Helsinki (IRB No.: NCC2014-0098).

### 2.2. Nutrient Intake

Nutrient intake was assessed using a validated semi-quantitative food frequency questionnaire consisting of 106 food items, frequencies of servings, and portion sizes. Daily nutrient intake values were estimated by the sum of the nutrients of each food item using the Food Composition Table of Korea [21,22]. Among the estimated nutrient intakes, total energy (kilocalories), protein, fat, carbohydrates (% of total energy), vitamin A, vitamin B_1_, vitamin B_2_, niacin, folate, vitamin B_6_, vitamin C, calcium, phosphorus, iron, and zinc were investigated in detail using the recommended dietary intakes established for Koreans [23].

### 2.3. Statistical Analysis

Of the matched participants, 1106 individuals had incomplete dietary questionnaires, including missing values for nutrient intake (48 cancer survivors, 595 non-cancer I, and 463 non-cancer II). After excluding these individuals, nutrient intake was compared between 5224 cancer survivors, 19,926 non-cancer I individuals, and 20,622 non-cancer II individuals using two methods: the mean intake of each nutrient between the three groups and the proportion of subjects with an intake of each nutrient higher than the recommended value compared to non-cancer I (reference group).

The baseline characteristics of the cancer survivors, non-cancer I individuals, and non-cancer II individuals were compared using the Chi-square test, and the mean of each nutrient level was compared by analysis of variance (ANOVA) with a post hoc comparison (Tukey’s test). Odds ratios (ORs) and 95% confidence intervals (CIs) were calculated to evaluate whether the cancer survivors and non-cancer individuals with comorbidities had nutrient intakes that were better than the intakes of the non-cancer I individuals by conditional logistic regression, adjusted for age, marital status, education level, income level, job status, smoking status, drinking status, current physical activity, and body mass index, based on measured height and weight. Although age was used as one of the matching variables, we also adjusted for age in the conditional logistic regression analysis due to the significant differences in age between the three groups. Body mass index was categorized as <23, 23–24.9, and ≥25 kg/m^2^ according to the report from the World Health Organization for Asia-Pacific regions [24,25]. Additionally, the Wald test was applied for differences in nutrient intakes between the cancer survivors and non-cancer II individuals. A subgroup analysis for each cancer type with 300 or more cases was conducted for the mean nutrient comparison, and the ORs were compared with non-cancer I. Subgroups with less than 300 cases were classified as an “other cancer” group. Cancer survivors diagnosed with more than one cancer type were classified as a “multiple cancer” group. The statistical software package SAS version 9.2 (SAS Institute, Cary, NC, USA) and R version 3.2.2 (R Foundation, Vienna, Austria) was used for all analyses.

## 3. Results

The mean age of the 5224 cancer survivors was 55.8 years, and 74.9% were women. The top five cancer types (stomach, colon, breast, cervix, and thyroid) accounted for 66% of the cancer survivors, and the mean interval from diagnosis was 6.6 years. Table 1 shows the baseline characteristics of study participants in three groups.

Table 2 shows the comparison of the means of each nutrient intake. The daily total energy, protein, proportion of total energy from protein, fat, proportion of total energy from fat intake, as well as vitamin B_1_, vitamin B_2_, niacin, phosphorus, and zinc intakes were lower in the cancer survivors than in the non-cancer I individuals. However, the proportion of total energy from carbohydrates, fiber and vitamin C intakes were higher compared to the non-cancer I individuals. When compared with the non-cancer II individuals, the cancer survivors’ protein, proportion of total energy from protein, fat, proportion of total energy from fat, and vitamin B_1_ intakes were lower, whereas the proportion of total energy from carbohydrates and the fiber, folate, vitamin C, vitamin E, iron, and potassium intakes were higher.

Table 3 shows the proportions and adjusted conditional ORs of the proportions of nutrient intakes that were higher than the recommended value for each nutrient. In all three groups, more than 80% of the subjects had a proportion of energy intake from carbohydrates that was higher than the recommended value and 1% or less of the subjects had a higher proportion of energy intake from fat than the recommended value. The cancer survivors consumed more energy from carbohydrates and less from fat.

The ORs for total energy, proportion of total energy from fat, and protein were significantly lower for the cancer survivors than they were for non-cancer I individuals (OR = 0.92, 95% CI = 0.86–0.99; OR = 0.54, 95% CI = 0.35–0.83; and OR = 0.85, 95% CI = 0.79–0.90, respectively), whereas the OR for the proportion of total energy from carbohydrates was higher (OR = 1.21, 95% CI = 1.10–1.33). Moreover, the cancer survivors’ protein, vitamin B_1_, vitamin B_2_, niacin, and phosphorus intakes were lower than the non-cancer I individuals. Conversely, the proportion of cancer survivors with a vitamin C intake above the recommended value was higher (OR = 1.16, 95% CI = 1.08–1.23). Compared with the non-cancer II individuals, the cancer survivors’ macronutrient intakes, including the proportions of total energy from fat intake and protein, were lower, whereas the proportion of total energy from carbohydrates and the vitamin A, folate, vitamin B_6_, vitamin C, and iron intakes were higher (*p* < 0.05, Table 3).

When we divided the cancer survivors by cancer type, the thyroid cancer survivors did not show significant differences in their intakes of any nutrients. However, the proportion of total energy from fat intake was lower and the proportion of total energy from carbohydrate intake was higher in all other cancer types than they were in the non-cancer I individuals. Additionally, the gastric cancer survivors’ protein, vitamin B_1_, vitamin B_2_, calcium, and phosphorus intakes were lower and the breast and cervical cancer survivors’ total energy, protein, and vitamin B_1_ intakes were lower (Table 4).

Table 5 shows the adjusted ORs for intakes higher than the recommended levels for each nutrient in each type of cancer survivor compared with the non-cancer I individuals. The ORs of the proportion of total energy from carbohydrates were significantly higher in the colon and breast cancer survivors, suggesting that these individuals consumed more carbohydrates than the non-cancer population. Additionally, protein intakes were lower in the gastric, breast, and cervical cancer survivors. The ORs of vitamin B_1_ intake were significantly lower in the gastric, colon, and breast cancer survivors, niacin intake ORs were lower in the breast cancer survivors, and phosphorus intake ORs were lower in the gastric, breast, and cervical cancer survivors. Conversely, for vitamin C intake, ORs were significantly higher in the gastric and colon cancer survivors.

## 4. Discussion

This study is one of the few studies to compare nutrient intake in cancer survivors with matched, unaffected people, based on population study results. The results suggested that cancer survivors’ nutrient intake statuses were poor, compared with non-cancer individuals without comorbidities, in terms of total energy, the proportion of energy from carbohydrates and fat, B vitamins, niacin, and phosphorus, but were better in regard to vitamin C intake. Even when compared with non-cancer individuals with comorbidities, the cancer survivors’ proportions of energy from carbohydrates and fat and their protein intakes were poor, although their micronutrient intakes were better.

Although several previous studies have reported the poor dietary intake of adult cancer survivors, these studies considered specific types of foods, such as only fruit and vegetable consumption (5-A-Day) [8,26], specific guideline adherence [27], or weight change as markers for nutrient intake [28]. One recent study conducted in a Western population estimated all nutrient intake levels and suggested poor adherence to dietary guidelines in cancer survivors, which was similar to our results. However, the nutrients with poor intakes differed between previous Western studies and this study. Zhang et al. showed higher energy intakes, saturated fat, and sodium and lower vitamin D, vitamin E, potassium, fiber, and calcium intakes in cancer survivors [13], which was in contrast to our results in which lower calorie, protein, and fat intakes were found. These different patterns of dietary nutrient intakes in cancer survivors may be affected by both different eating patterns in cancer survivors and background nutrient intake patterns in the general Western and Asian populations. The dietary protein, carbohydrate, saturated fat, B vitamin, niacin, and folate intakes were higher than the recommended levels in people without a history of cancer in the study by Zhang et al. [13], whereas the intakes of these nutrients were lower (with the exception of the carbohydrate intake) in this study population, even in the non-cancer individuals.

In Asian countries where polished rice-based meals are common, the diet is higher in carbohydrates but low in animal fat [29]. This difference may affect the lower levels of nutrient intake from animal products, such as fat, protein, and B vitamins. The mean values of all nutrients were comparable with previous nationally representative studies in Korea [30,31], suggesting that carbohydrate intake was higher and intake of nutrients from animal products was generally lower than recommended in Korea. Furthermore, several other studies conducted in Asian countries showed lower nutrient intake levels, especially for total energy, protein, and fat, in both the general population [32] and cancer survivors [33,34,35].

When we focused on the comparison of nutrient intakes in cancer survivors and non-cancer individuals, fat, protein, vitamin A, B vitamins, niacin, and phosphorus, which are primarily ingested from animal food sources, showed significantly lower intakes in the cancer survivors. The proportion of total energy from fat and the protein intake were even lower than the intakes in non-cancer II individuals, who generally require dietary restrictions due to comorbidities. Cancer survivors are highly motivated to adopt healthy lifestyle behaviors [7,36], and their interest in a healthy lifestyle has been shown to mostly focus on diet, especially a low-fat diet [37]. The nutrition guidelines for cancer survivors in Western countries generally recommend diets high in vegetables, fruits, and whole grains and low in fat [9]. However, for cancer survivors in Asian countries, such recommendations might make them increase their carbohydrate intake and reduce their intake of animal products, inducing far lower levels of protein and fat as proportions of the total caloric intake and relatively higher carbohydrate intakes. The higher vitamin C intake levels in survivors could suggest a higher intake of vegetables and fruits in cancer survivors, which follows the current guidelines well. Therefore, the adaptation of nutrient intake guidelines from Western countries without considering the differences in dietary intakes between Western and Asian countries in the general population may produce negative effects on nutrient intakes in cancer survivors.

To the best of our knowledge, few studies have investigated the differences in nutrient intakes between survivors by cancer type. Survivors of gastric, colon, breast, cervical, and thyroid cancer, which are the five major prevalent cancers in Korea [3], were compared with non-cancer I individuals. The intake patterns of survivors of thyroid cancer, which is the most common cancer in Korea, were similar or better than the non-cancer I individuals. However, gastric, breast, and cervical cancer survivors showed worse nutrient intake qualities with lower protein intake. Additionally, gastric and breast cancer survivors showed poor nutrient intakes for several micronutrients and minerals. The nutrient intakes of gastric cancer patients generally deteriorate due to gastrectomy and chemotherapy [38], which was supported by our results. However, a previous study in the United States proposed that breast cancer survivors have the best diet quality among the major types of cancer [13], indicating that a regional approach to nutrient intake was needed.

Although our study has strengths, several limitations should be considered. First, the cross-sectional design based on baseline measurements of a cohort prevented the assessment of changes or trends in dietary intake after cancer diagnosis in cancer survivors. Thus, we could not investigate the changes in nutrient intake before and after cancer diagnosis in cancer survivors. Second, the cancer survivors and non-cancer individuals with/without comorbidities were divided based on self-reports, which could cause misclassification. However, previous studies reported a reasonably high validity for self-reported cancer histories [39], and we considered the risk for misclassification bias to be minimal. Additionally, because the cancer stage and treatment information was not accessed by the questionnaires, evaluating their effects on dietary intakes in cancer survivors was not possible. Third, the subjects were recruited from health examination centers. Thus, they might be healthier than general cancer survivors or people without cancer and more concerned about healthy lifestyle, limiting the ability to generalize the results. Fourth, although we matched cancer survivors with non-cancer groups by age, gender, and recruitment center to eliminate their effects on dietary intake, the age distribution was still significantly different. We included age as one of the covariates in the conditional logistic regression model to reduce its effect. Additionally, we confirmed that the results were not significantly different, with the exception of minor changes in point estimates based on both the conditional analysis and logistic regression analysis (data not shown). Fifth, although food frequency questionnaires have been widely applied to access dietary intake in epidemiological studies, the measurement errors including the portion size or lower correlation with other tools, especially in Asian countries, should be considered [40]. Despite these limitations, to the best of our knowledge, this study is the first study to evaluate nutrient intake in cancer survivors as a whole and by cancer type compared with the non-cancer population using a relatively large sample size and a validated food frequency questionnaire. To increase the comparability, cancer survivors and two non-cancer groups were matched by age, gender, and recruitment center, and numerous possible covariates were adjusted for in the analysis. Additionally, the nutrient intake was compared with non-cancer individuals with comorbidities to obtain a perspective of cancer as a chronic disease.

## 5. Conclusions

A healthy diet is important for decreasing cancer-related adverse sequelae, comorbidities, and death and improving quality of life [7,28]. We observed poor nutrient intakes in the cancer survivors, especially a lower calorie intake, inappropriate proportions of total energy (higher proportion of carbohydrate and lower proportion of fat and protein), and lower micronutrients derived from animal products. This pattern was different from observations in Western countries, where the higher intakes of energy and fat are important issues. Therefore, the adaptation of nutrition guidelines in Western countries for cancer survivors in Asia needs to be re-considered, and guidelines for Asian cancer survivors should be established based on dietary intake patterns in the general population.

## Figures and Tables

**Table 1:** Baseline characteristics of cancer survivors, non-cancer individuals without comorbidities (non-cancer I), and non-cancer individuals with comorbidities (non-cancer II).

Characteristics	*N* (%)			*p*-Value
	Non-Cancer I (*N* = 19,926)	Non-Cancer II (*N* = 20,622)	Cancer Survivors (*N* = 5224)	
Age				
Mean ± Standard deviation	54.6 (8.0)	55.8 (8.1)	55.8 (8.1)	<0.001
<50	5674 (28.5)	4851 (23.5)	1256 (24.0)	<0.001
50–54	4415 (22.2)	4325 (21.0)	1098 (21.0)	
55–59	3826 (19.2)	4105 (19.9)	1014 (19.4)	
60–64	3447 (17.3)	3917 (19.0)	964 (18.5)	
≥65	2564 (12.9)	3424 (16.6)	892 (17.1)	
Gender				
Male	5010 (25.1)	5169 (25.1)	1309 (25.1)	0.98
Female	14,916 (74.9)	15,453 (74.9)	3915 (74.9)	
Marital status				
Married, cohabitant	17,473 (87.7)	17,919 (86.9)	4536 (86.8)	0.002
Others	2307 (11.6)	2617 (12.7)	671 (12.8)	
Missing	146 (0.7)	86 (0.4)	17 (0.3)	
Education				
<High school	7240 (36.3)	8266 (40.1)	1954 (37.4)	<0.001
High school graduate	7152 (35.9)	7034 (34.1)	1870 (35.8)	
≥College	5174 (26)	5062 (24.5)	1344 (25.7)	
Missing	360 (1.8)	260 (1.3)	56 (1.1)	
Monthly household income				
<$2000	5917 (29.7)	6697 (32.5)	1788 (34.2)	<0.001
$2000–3999	7210 (36.2)	6960 (33.8)	1776 (34.0)	
≥$4000	3743 (18.8)	3769 (18.3)	949 (18.2)	
Missing	3056 (15.3)	3196 (15.5)	711 (13.6)	
Employment status				
Employed	9547 (47.9)	8722 (42.3)	1987 (38)	<0.001
Unemployed	9891 (49.6)	11,340 (55)	3131 (59.9)	
Missing	488 (2.4)	560 (2.7)	106 (2.0)	
Smoking status				
Never	15,898 (79.8)	16,310 (79.1)	4112 (78.7)	<0.001
Past	2171 (10.9)	2594 (12.6)	816 (15.6)	
Current	1752 (8.8)	1643 (8)	276 (5.3)	
Missing	105 (0.5)	75 (0.4)	20 (0.4)	
Drinking status				
Never	11,267 (56.5)	11,780 (57.1)	3254 (62.3)	<0.001
Past	537 (2.7)	864 (4.2)	523 (10.0)	
Current	8006 (40.2)	7905 (38.3)	1431 (27.4)	
Missing	116 (0.6)	73 (0.4)	16 (0.3)	
Regular exercise				
No	9662 (48.5)	9507 (46.1)	2187 (41.9)	<0.001
<150 min/week	2976 (14.9)	3093 (15.0)	714 (13.7)	
≥150 min/week	7193 (36.1)	7979 (38.7)	2316 (44.3)	
Missing	95 (0.5)	43 (0.4)	7 (0.1)	
Body mass index				
<23 kg/m^2^	8799 (44.2)	7511 (36.4)	2285 (43.7)	<0.001
23–24.9 kg/m^2^	5657 (28.4)	5725 (27.8)	1384 (26.5)	
≥25 kg/m^2^	5422 (27.2)	7327 (35.5)	1537 (29.4)	
Missing	48 (0.2)	59 (0.3)	18 (0.3)	

**Table 2:** Nutrient intake in cancer survivors, non-cancer individuals without comorbidities (non-cancer I), and non-cancer individuals with comorbidities (non-cancer II).

Nutrient	Mean (Standard Deviation)			*p*-Value
	Non-Cancer I (*N* = 19,926)	Non-Cancer II (*N* = 20,622)	Cancer Survivors (*N* = 5224)	
Macronutrients				
Total energy, kcal	1739.1 (576.2)	1713.3 (589.9) ^a^	1699.2 (559.4) ^a^	<0.001
Protein, g	59.4 (26.9)	58.2 (26.9) ^a^	57.2 (24.7) ^a,b^	<0.001
% Energy	13.5 (2.7)	13.4 (2.7) ^a^	13.3 (2.6) ^a,b^	<0.001
Fat, g	27.6 (18.3)	26.5 (18.5) ^a^	25.0 (16.3) ^a,b^	<0.001
% Energy	13.8 (5.5)	13.4 (5.5) ^a^	12.8 (5.3) ^a,b^	<0.001
Carbohydrate, g	309.2 (94.4)	306.5 (97.0) ^a^	307.8 (95.6)	0.02
% Energy	71.7 (7.1)	72.2 (7.1) ^a^	73.0 (6.9) ^a,b^	<0.001
Fiber	5.9 (3.1)	5.8 (3.1)	6.0 (3.0) ^a,b^	<0.001
Micronutrients				
Vitamin A, R.E.	496.5 (375.2)	486.0 (373.7) ^a^	497.0 (353.7)	0.009
Vitamin B_1_, mg	1.0 (0.5)	1.0 (0.5) ^a^	1.0 (0.4) ^a,b^	<0.001
Vitamin B_2_, mg	0.9 (0.5)	0.9 (0.5) ^a^	0.9 (0.4) ^a^	<0.001
Niacin, mg	14.6 (6.5)	14.1 (6.5) ^a^	14.0 (6.0) ^a^	<0.001
Folate, µg	223.0 (129.5)	220.7 (131.7)	227.3 (126.0) ^b^	0.004
Vitamin B_6_, mg	1.6 (0.7)	1.6 (0.7) ^a^	1.6 (0.7)	0.001
Vitamin C, mg	110.2 (73.0)	109.2 (74.3)	114.1 (74.1) ^a,b^	<0.001
Vitamin E, mg	8.2 (4.7)	8.1 (4.8) ^a^	8.3 (4.6) ^b^	0.009
Minerals				
Calcium, mg	458.1 (281.5)	451.4 (279.9) ^a^	454.0 (269.1)	0.049
Phosphorus, mg	900.4 (378.6)	883.0 (379.8) ^a^	876.6 (357.0) ^a^	<0.001
Iron, mg	10.1 (5.3)	10.0 (5.3) ^a^	10.2 (5.2) ^b^	0.01
Potassium, mg	2292.3 (1117.5)	2246.1 (1132.4) ^a^	2283.6 (1101.9) ^b^	<0.001
Zinc, µg	8.0 (3.9)	7.9 (4.1) ^a^	7.8 (3.6) ^a^	0.006

^a^ *p* < 0.05 compared with non-cancer individuals without comorbidities in a multiple comparison test (Tukey’s test); ^b^ *p* < 0.05 compared with non-cancer individuals with comorbidities in the multiple comparison test (Tukey’s test).

**Table 3:** Proportion and conditional logistic regression for higher dietary nutrient intake than recommended in cancer survivors and non-cancer individuals with comorbidities (non-cancer II) compared with non-cancer individuals without comorbidities (non-cancer I).

Nutrient	Higher Dietary Nutrient Intake Than Recommended Level, *N* (%)			Odds Ratio ^a^ (95% Confidence Interval (CI))			*p*-Value ^b^
	Non-Cancer I (*N* = 19,926)	Non-Cancer II (*N* = 20,622)	Cancer Survivors (*N* = 5224)	Non-Cancer I	Non-Cancer II	Cancer Survivors	
Total energy	6392 (32.1)	6438 (31.2)	1608 (30.8)	Reference	0.93 (0.89–0.98)	0.92 (0.86–0.99)	0.81
Carbohydrate (% of total energy)	16901 (84.8)	17649 (85.6)	4585 (87.8)		1.01 (0.96–1.08)	1.21 (1.10–1.33)	<0.001
Fat (% of total energy)	203 (1.0)	184 (0.9)	26 (0.5)		0.90 (0.73–1.13)	0.54 (0.35–0.83)	0.02
Protein	11117 (55.8)	11046 (53.6)	2696 (51.6)		0.90 (0.86–0.94)	0.85 (0.79–0.90)	0.05
Vitamin A	4429 (22.2)	4459 (21.6)	1207 (23.1)		0.94 (0.89–0.99)	1.02 (0.95–1.11)	0.02
Vitamin B_1_	5871 (29.5)	5670 (27.5)	1349 (25.8)		0.92 (0.88–0.97)	0.86 (0.80–0.93)	0.08
Vitamin B_2_	3395 (17.0)	3290 (16.0)	802 (15.4)		0.94 (0.89–1.00)	0.89 (0.81–0.97)	0.22
Niacin, mg	8206 (41.2)	7893 (38.3)	1976 (37.8)		0.90 (0.87–0.94)	0.91 (0.85–0.97)	0.75
Folate	1575 (7.9)	1535 (7.4)	441 (8.4)		0.93 (0.86–1.01)	1.05 (0.93–1.18)	0.04
Vitamin B_6_	10627 (53.3)	10624 (51.5)	2775 (53.1)		0.93 (0.89–0.97)	1.01 (0.96–1.08)	0.01
Vitamin C	9156 (46.0)	9347 (45.3)	2589 (49.6)		0.98 (0.94–1.03)	1.16 (1.08–1.23)	<0.001
Calcium	2162 (10.9)	2033 (9.9)	564 (10.8)		0.89 (0.83–0.96)	0.96 (0.87–1.07)	0.15
Phosphorus	13873 (69.6)	13802 (66.9)	3434 (65.7)		0.89 (0.86–0.94)	0.86 (0.81–0.92)	0.22
Iron	9019 (45.3)	9579 (46.5)	2504 (47.9)		0.98 (0.94–1.03)	1.05 (0.98–1.13)	0.045
Zinc	8714 (43.7)	8823 (42.8)	2233 (42.7)		0.94 (0.90–0.98)	0.96 (0.89–1.02)	0.65

^a^ Analyses were adjusted for age, marital status, education level, income level, job status, smoking status, drinking status, current physical activity, and body mass index; ^b^ Differences between non-cancer individuals with comorbidities and cancer survivors in the Wald test.

**Table 4:** Dietary intake in cancer survivors according to cancer type.

Nutrient	Mean (Standard Deviation)						
	Gastric Cancer	Colon Cancer	Breast Cancer	Cervical Cancer	Thyroid Cancer	Other Cancer	Multiple Cancers
	*N* = 727	*N* = 372	*N* = 864	*N* = 603	*N* = 880	*N* = 1405	*N* = 136
Macronutrients							
Total energy, kcal	1681.3 (552.8)	1704.1 (531.1)	1662.1 (55.85) ^a^	1661.5 (541.4) ^a^	2734.4 (587.4)	1726.5 (561.4)	1628.1 (522.7)
Protein, g	55.9 (25.5) ^a^	57.3 (24.8)	55.2 (22.9) ^a^	56.0 (24.1) ^a^	58.8 (25.0)	58.6 (25.5)	53.9 (22.3)
% Energy	13.1 (2.7) ^a^	13.3 (2.5)	13.2 (2.5)	13.4 (2.7)	13.4 (2.5)	13.4 (2.8)	13.1 (2.4)
Fat, g	24.6 (18.2) ^a^	24.4 (15.8) ^a^	22.9 (14.3) ^a^	24.8 (15.4) ^a^	26.9 (17.0)	25.9 (16.4) ^a^	21.6 (13.2) ^a^
% Energy	12.5 (5.5) ^a^	12.4 (4.8) ^a^	12.2 (5.0) ^a^	13.0 (5.3) ^a^	13.5 (5.1)	13.0 (5.5) ^a^	11.5 (5.1) ^a^
Carbohydrate, g	305.2 (90.9)	310.2 (88.8)	306.0 (100.9)	300.4 (93.7)	310.8 (98.5)	311.0 (95.4)	301.5 (94.0)
% Energy	73.4 (7.2) ^b^	73.4 (6.5) ^b^	73.9 (6.6) ^b^	72.7 (6.9) ^b^	72.2 (6.6)	72.6 (7.2) ^b^	74.5 (6.4) ^b^
Fiber	5.8 (3.0)	6.1 (3.0)	6.2 (3.1) ^a^	5.9 (3.0)	6.0 (3.0)	6.1 (3.1)	6.1 (3.2)
Micronutrients							
Vitamin A, R.E.	478.7 (356.8)	497.2 (338.3)	502.4 (353.1)	483.4 (341.6)	485.4 (331.9)	511.7 (367.1)	51.89 (401.1)
Vitamin B_1_, mg	0.93 (0.48) ^a^	0.95 (0.39)	0.92 (0.40) ^a^	0.94 (0.41) ^a^	0.97 (0.42)	0.98 (0.42)	0.91 (0.42)
Vitamin B_2_, mg	0.82 (0.43) ^a^	0.88 (0.43)	0.88 (0.42)	0.88 (0.42)	0.91 (0.45)	0.91 (0.45)	0.83 (0.46)
Niacin, mg	13.8 (6.4)	13.9 (5.6)	13.5 (5.6)	13.6 (6.0)	14.4 (5.8)	14.4 (6.2)	13.2 (5.7)
Folate, µg	217.7 (122.3)	226.1 (124.3)	233.8 (127.1)	223.7 (122.4)	223.9 (120.7)	231.4 (128.8)	232.7 (153.2)
Vitamin B_6_, mg	1.6 (0.7)	1.6 (0.7)	1.6 (0.7)	1.6 (0.7)	1.6 (0.7)	1.6 (0.7)	1.6 (0.7)
Vitamin C, mg	105.2 (66.3)	109.8 (63.1)	120.7 (76.9)	113.7 (74.8)	114.3 (71.2)	115.0 (76.0)	120.8 (105.1)
Vitamin E, mg	7.9 (4.3)	8.1 (4.2)	8.3 (4.5)	8.1 (4.5)	8.4 (5.0)	8.4 (4.7)	7.9 (4.7)
Minerals							
Calcium, mg	405.0 (243.9) ^a^	443.1 (264.5)	463.4 (266.7)	456.0 (255.2)	475.1 (281.2)	462.0 (275.8)	444.6 (294.4)
Phosphorus, mg	843.4 (349.6) ^a^	871.0 (353.0)	861.3 (340.3)	865.2 (344.4)	903.3 (366.4)	893.2 (367.6)	842.9 (359.5)
Iron, mg	9.9 (5.2)	10.2 (5.0)	10.3 (5.2)	9.9 (4.8)	10.3 (5.0)	10.4 (5.4)	10.0 (5.4)
Potassium, mg	2157.1 (1050.3)	227.5 (1046.9)	2313.2 (1100.0)	2251.5 (1084.9)	2306.9 (1100.7)	2326.4 (1128.2)	2262.2 (1248.3)
Zinc, µg	7.6 (3.4)	7.9 (3.2)	7.6 (3.4)	7.6 (3.3)	7.9 (3.1)	8.1 (4.0)	7.6 (3.1)

^a^ Significantly lower than non-cancer individuals without comorbidities in the multiple comparison test (Tukey’s test); ^b^ Significantly higher than non-cancer individuals without comorbidities in the multiple comparison test (Tukey’s test).

**Table 5:** Conditional logistic regression for higher dietary nutrient intakes than recommended in survivors of each type of cancer compared with non-cancer individuals without comorbidities (non-cancer I).

Nutrient	Non-Cancer I *N* = 19,926	Odds Ratio ^a^ (95% Confidence Interval)						
		Gastric Cancer	Colon Cancer	Breast Cancer	Cervical Cancer	Thyroid Cancer	Other Cancer	Multiple Cancers
		*N* = 727	*N* = 372	*N* = 864	*N* = 603	*N* = 880	*N* = 1405	*N* = 136
Total energy	Reference	0.83 (0.69–1.02)	0.82 (0.62–1.08)	0.89 (0.76–1.05)	0.90 (0.74–1.09)	0.94 (0.80–1.11)	0.98 (0.86–1.11)	0.85 (0.56–1.30)
Carbohydrate (% of total energy)		1.23 (0.95–1.61)	1.50 (1.03–2.18)	1.83 (1.40–2.39)	0.87 (0.66–1.13)	1.15 (0.92–1.43)	1.08 (0.92–1.28)	1.83 (0.93–3.60)
Fat (% of total energy)		- ^b^	- ^b^	- ^b^	- ^b^	- ^b^	- ^b^	- ^b^
Protein		0.76 (0.64–0.91)	0.88 (0.69–1.11)	0.79 (0.67–0.93)	0.78 (0.65–0.95)	1.03 (0.87–1.20)	0.85 (0.76–0.96)	0.72 (0.48–1.07)
Vitamin A		1.01 (0.81–1.26)	1.13 (0.85–1.52)	1.03 (0.86–1.23)	0.96 (0.77–1.20)	0.95 (0.79–1.14)	1.14 (0.99–1.30)	0.90 (0.58–1.39)
Vitamin B_1_		0.79 (0.64–0.97)	0.76 (0.64–0.99)	0.75 (0.62–0.89)	0.81 (0.66–1.01)	0.97 (0.82–1.15)	0.95 (0.84–1.08)	0.66 (0.41–1.04)
Vitamin B_2_		0.84 (0.64–1.10)	0.77 (0.53–1.12)	0.83 (0.68–1.02)	0.89 (0.70–1.14)	0.94 (0.76–1.15)	1.02 (0.87–1.20)	0.53 (0.29–0.97)
Niacin, mg		0.90 (0.75–1.08)	1.01 (0.78–1.29)	0.76 (0.65–0.90)	0.87 (0.71–1.05)	0.96 (0.82–1.13)	0.96 (0.85–1.08)	0.66 (0.44–1.01)
Folate		1.10 (0.79–1.54)	0.90 (0.49–1.31)	0.93 (0.70–1.23)	0.99 (0.70–1.38)	1.14 (0.85–1.51)	1.25 (1.02–1.54)	1.09 (0.58–2.04)
Vitamin B_6_		1.01 (0.85–1.20)	1.12 (0.88–1.42)	0.94 (0.80–1.10)	0.92 (0.77–1.12)	1.15 (0.98–1.35)	0.98 (0.87–1.10)	0.84 (0.57–1.25)
Vitamin C		1.25 (1.05–1.49)	1.28 (1.01–1.63)	1.15 (0.98–1.34)	1.17 (0.97–1.42)	1.08 (0.92–1.26)	1.19 (1.05–1.33)	1.01 (0.68–1.51)
Calcium		0.79 (0.59–1.08)	0.91 (0.60–1.37)	0.79 (0.62–1.02)	0.99 (0.73–1.35)	1.27 (1.00–1.60)	1.08 (0.90–1.30)	0.84 (0.44–1.63)
Phosphorus		0.78 (0.65–0.94)	0.83 (0.65–1.07)	0.81 (0.68–0.96)	0.76 (0.63–0.93)	1.09 (0.92–1.29)	0.85 (0.75–0.96)	0.73 (0.49–1.11)
Iron		1.04 (0.87–1.25)	1.05 (0.82–1.34)	1.04 (0.88–1.24)	0.99 (0.81–1.21)	1.18 (1.00–1.40)	1.03 (0.91–1.16)	0.86 (0.57–1.29)
Zinc		0.79 (0.65–0.95)	0.91 (0.70–1.17)	0.93 (0.79–1.09)	0.95 (0.78–1.14)	1.08 (0.92–1.26)	0.99 (0.88–1.11)	0.67 (0.45–1.01)

^a^ Analyses were adjusted for age, marital status, education level, income level, job status, smoking status, drinking status, current physical activity, and body mass index; ^b^ Analyses were impossible due to the small number of survivors of each cancer type with a higher proportion of the total caloric intake from fat (1 to 10).
